# Regulation of CXCR6 Expression on Adipocytes and Osteoblasts Differentiated from Human Adipose Tissue-Derived Mesenchymal Stem Cells

**DOI:** 10.1155/2020/8870133

**Published:** 2020-08-19

**Authors:** Seung-Cheol Lee, Yoo-Jung Lee, Min Kyoung Shin, Jung-Suk Sung

**Affiliations:** Department of Life Science, Dongguk University-Seoul, Goyang, Gyeonggi-do 10326, Republic of Korea

## Abstract

Human mesenchymal stem cells derived from adipose tissue (hADMSCs) are a desirable candidate in regenerative medicine. hADMSCs secrete growth factors, cytokines, and chemokines and also express various receptors that are important in cell activation, differentiation, and migration to injured tissue. We showed that the expression level of chemokine receptor CXCR6 was significantly increased by ~2.5-fold in adipogenic-differentiated cells (Ad), but not in osteogenic-differentiated cells (Os) when compared with hADMSCs. However, regulation of CXCR6 expression on hADMSCs by using lentiviral particles did not affect the differentiation potential of hADMSCs. Increased expression of CXCR6 on Ad was mediated by both receptor recycling, which was in turn regulated by secretion of CXCL16, and de novo synthesis. The level of soluble CXCL16 was highly increased in both Ad and Os in particular, which inversely correlates with the expression on a transmembrane-bound form of CXCL16 that is cleaved by disintegrin and metalloproteinase. We concluded that the expression of CXCR6 is regulated by receptor degradation or recycling when it is internalized by interaction with CXCL16 and by de novo synthesis of CXCR6. Overall, our study may provide an insight into the molecular mechanisms of the CXCR6 reciprocally expressed on differentiated cells from hADMSCs.

## 1. Introduction

Human mesenchymal stem cells derived from adipose tissue (hADMSCs) are multipotent stem cells that can be differentiated into multiple lineages such as adipocyte, osteoblasts, chondrocytes, and neuronal cells [1, 2]. hADMSCs are widely used for regenerative medicine due to their significant therapeutic capability. Previous studies demonstrated that hADMSCs can migrate to the injured tissue areas in response to homing signals to repair the damaged tissues [3–5]. It has been known that migration of hADMSCs is regulated by the secretion of various growth factors, cytokines, and chemokines or activation of their receptors [5, 6].

Chemokine receptors are considered as promising drug targets since they regulate many biological processes such as inflammation, infection, cancer metastasis, and angiogenesis [7, 8]. Among many chemokine receptors, CCR1, CXCR4, CCR7, CXCR6, and CXCR3 have been known to play important roles in cell migration and recruitment in different types of leukocytes or stem cells, through their binding with specific chemokines [9–12]. Of interests, while the chemokine receptor in the stem cells including their functional roles in cell migration has been intensively studied, chemokine receptors in the differentiated cells from hADMSCs have not yet been investigated.

CXC-motif receptor (CXCR) 6 is a member of the chemokine receptors interacting with the single chemokine CXCL16 leading to the recruitment of T cells that induce the chronic inflammation in rheumatoid arthritis synovia [13, 14]. CXCL16 as a transmembrane-bound form is a scavenger receptor for phosphatidylserine or oxidized lipoproteins. It is proteolytically cleaved by ADAMs (abbreviation for a disintegrin and metalloproteinase) which are a family of transmembrane and secreted metalloproteinase [15]. Upon the cleavage, it is subsequently secreted as a soluble molecule that induces chemotaxis of CXCR6 expressing T, natural killer, and natural killer T cells to sites of inflammation and injury [16]. ADAM10 is the major protease responsible for cleavage of the scavenger receptor to a soluble chemoattractant that subsequently binds to its specific receptor [17–20].

The number of chemokine receptor on the cell surface is important to maintain homeostasis, which is regulated by receptor internalization by endocytosis in response to prolonged interaction with specific chemokines [21, 22]. When the chemokine receptor is internalized, recruited *β*-arrestin2 binds to it and its fate is determined by which protein (chemokine receptor or *β*-arrestin2) is polyubiquitinated [23–26]. Ubiquitination of the chemokine receptor leads to its dissociation from *β*-arrestin2, and this may result in receptor degradation. Alternatively, chemokine receptor is recycled back to the plasma membrane by ubiquitination of *β*-arrestin2 [21]. The level of chemokine receptor on the cell surface is also regulated by its transportation to the cell surface through late endosomes and the Golgi apparatus as shown to play a role in the replenishment of chemokine receptor [25]. We hypothesized that this study on the regulation of its level may lead to elucidate the mechanism of CXCR6 expression on the surface of hADMSCs and differentiated cells.

Here, it is the first investigation on demonstrating the differential expression of CXCR6 on cell surfaces is regulated by receptor degradation or recycling and also by de novo synthesis of a receptor by CXCL16 stimulation. Our study may provide basic information on the molecular mechanisms of CXCR6 expression that may have a potential role in regulating cellular functions during hADMSC differentiation into adipocytes.

## 2. Materials and Methods

### 2.1. Cell Culture and Adipogenic or Osteogenic Differentiation Induction

hADMSCs were purchased from CEFO (Korea) and cultured in hADMSC growth medium (CEFO) for 6 days. Experiments were performed after detachment of cells by Accutase (Innovative Cell Technologies, USA), and then, experiments were performed. For adipogenic and osteogenic differentiation, biological passage number 4 was used. Adipogenic-differentiated cells (Ad) were induced by culturing cells for 12 days and 18 days in high-glucose Dulbecco's Modified Eagle's Medium (DMEM) (Gibco, USA) containing with 1% penicillin-streptomycin (Gibco), 10% fetal bovine serum (FBS; Alphabioregen, USA), 1 *μ*M dexamethasone (Sigma, USA), 100 *μ*M indomethacin (Sigma), 10 *μ*g/ml insulin (Welgene, Korea), and 500 *μ*M 3-isobutyl-1-methylxanthine (Sigma). Osteogenic-differentiated cells (Os) were also induced by culturing cells for 12 days and 18 days in osteogenic differentiation medium containing low-glucose DMEM (Gibco), 1% penicillin-streptomycin (Gibco), 10% FBS, 0.1 *μ*M dexamethasone (Sigma), 10 mM *β*-glycerophosphate (Sigma), and 50 *μ*M L-ascorbic acid-2-phosphate (Sigma).

### 2.2. Oil Red O (ORO) Staining

Adipogenic differentiation was evaluated by ORO staining of triglyceride vesicles. Cells were washed twice with PBS and fixed with 10% formalin (Sigma) for 10 min. After fixation, cells were washed with PBS and 60% isopropanol (Sigma) and dried. ORO (Sigma) staining was performed at room temperature for 30 min. Then, cells were washed twice with distilled water and imaged by microscopy (Nikon, Japan). To quantify the remaining ORO-stained on cells, the dye was dissolved by 100% isopropanol and the absorbance was measured at 492 nm by an enzyme-linked immunosorbent assay (ELISA) reader (Tecan, Switzerland).

### 2.3. Alizarin Red S (ARS) Staining

Osteogenic differentiation was evaluated by ARS staining. Cells were washed twice with DPBS and fixed with 10% formalin (Sigma) for 15 min. After fixation, cells were washed with DPBS and then treated with ARS (Sigma) staining solution for 45 min at room temperature in the dark. Next, cells were washed four times with distilled water and imaged by microscopy (Nikon). To quantify the remaining ARS-stained on cells, the dye was dissolved by 10% cetylpyridinium chloride (Sigma) and the absorbance was measured at 570 nm by an ELISA reader.

### 2.4. Flow Cytometry

Cells were harvested with cell dissociation buffer (Gibco) and centrifuged at 1,200 rpm for 5 min and blocked with 1% bovine serum albumin (BSA, Sigma) for 30 min and then stained with BV421 mouse anti-human CXCR6 (1: 100), BV421 mouse IgG2a, and k isotype control (BD Biosciences) at 4°C for 45 min. Stained cells were centrifuged at 4,000 rpm for 5 min and fixed in 2% paraformaldehyde for 10 min, then analyzed by flow cytometry using a BD FACSCanto™ flow cytometer (BD Biosciences) after resuspension in FACS buffer. Data were analyzed with BD FACSDiva v8.0 software.

### 2.5. Immunocytochemistry (ICC)

Cells were fixed with 4% paraformaldehyde to detect the cell membrane protein and blocked with 1% BSA for 30 min. Cells were then incubated with CXCR6 (GeneTex, USA) or CXCL16 (GeneTex) primary antibodies at 4°C overnight. To detect cells stained by primary antibodies, the cells were incubated with Alexa 488-conjugated anti-rabbit secondary antibodies (Cell Signaling, USA) for 45 min. Nuclei were counterstained with 4,6-diamidino-2-phenylindole (DAPI). Fluorescent images were obtained by confocal microscopy (Nikon) and quantitatively analyzed by the ImageJ program (National Institutes of Health, USA).

### 2.6. RNA Extraction and Real-Time PCR

RNA was isolated using Trizol (Invitrogen, USA) as per the manufacturer's protocol. With 0.5 *μ*g of total RNA as a template, cDNA was synthesized by using the Reverse Transcription Master Premix (ELPIS, Korea) according to the manufacturer's instructions. To amplify cDNA, 40 cycles of PCR were performed by using the SYBR Green PCR Master Mix (KAPA Biosystem, USA) according to the manufacturer's protocol. The expression level of each target gene was normalized to internal control, *GAPDH*.

### 2.7. *CXCR6* Knockdown

Knockdown of *CXCR6* in hADMSCs was achieved with *CXCR6* shRNA lentiviral particles according to the manufacturer's protocol (Santa Cruz, USA). Briefly, hADMSCs were plated onto 6-well plates, 2 days before transduction to 70% confluence. Cells were transduced with either 8 *μ*l of CXCR6 shRNA lentiviral particles (Santa Cruz) or control shRNA (scramble) lentiviral particles-A (Santa Cruz) per well in Opti-MEM (Gibco) plus 8 *μ*g/ml polybrene (Sigma) and centrifuged at 200 x *g* for 2 h. Medium with lentiviral particle was removed and changed to high-glucose DMEM (Gibco) in 10% FBS the next day. Two days after transduction, the cells were subjected to puromycin selection (4 *μ*g/ml) for 6 days. After puromycin treatment, no viable cells were observed in the well containing mock-transduced cells. The transduced cells, puromycin resistant, were collected, to examine the knockdown efficiency or for induction to adipogenic or osteogenic differentiation. The efficiency of CXCR6 knockdown was assessed at the gene expression level by RT-PCR analysis and at the protein level by immunoblotting analysis. Cells transduced with CXCR6 shRNA and scramble shRNA lentiviral particles are referred to as sh-CXCR6 and sh-control, respectively.

### 2.8. *CXCR6* Overexpression

CXCR6 lentiviral particle and control lentiviral particle were obtained by the CXCR6 lentiviral vector (abm, Canada) and lentiviral control vector (Cell Biolabs, USA) with viral packaging mix (abm) according to the manufacturer's protocol, respectively. The obtained supernatant of lentiviral particle was concentrated by using a Lenti-X concentrator (Clontech, USA), as per the manufacturer's protocol. The next procedure was performed the same as that of sh-CXCR6 lentiviral particles. Cells transduced with CXCR6 lentiviral vector and control lentiviral vector are referred to as LV-CXCR6 and LV-control, respectively.

### 2.9. Western Blot

Cells were lysed using RIPA buffer (Biosolution, Korea), phenylmethylsulfonyl fluoride (Sigma), protease inhibitor, and phosphatase inhibitor cocktail 2/3 (Sigma) and then centrifuged at 25,000 x *g* for 30 min. Cell extracts were quantified using the BCA protein assay kit (Pierce Biotechnology, USA) according to the manufacturer's instructions. The proteins (20-30 *μ*g) were separated on 10% sodium dodecyl sulfate-polyacrylamide gel electrophoresis (SDS-PAGE) and transferred to polyvinylidene difluoride membranes (GE Healthcare, UK) for western blot analyses. The transferred membrane was blocked with 1x Tris-buffered saline with Tween 20 (Sigma) (TBST) containing 5% skim milk (BD Biosciences) for 1 h and then incubated with primary antibodies ADAM10 (Santa Cruz), ADAM17 (Abcam, USA), *β*-arrestin (Bethyl, USA), and CXCR6 (GeneTex) in 1x TBST containing 1% skim milk at 4°C overnight. The membrane was washed three times with 1x TBST for 10 min, then incubated with secondary anti-rabbit (Cell Signaling) and anti-mouse (Santa Cruz) antibodies in 1x TBST containing 1% skim milk for 45 min. The membrane was washed three times with 1x TBST for 15 min and was visualized with enhanced chemiluminescence detection reagent (Amersham Pharmacia Biotech, USA) and imaged by ChemiDoc (Bio-Rad Laboratories, USA).

### 2.10. Immunoprecipitation

Cells were lysed with 1 M Tris-HCl (Sigma), 1 M NaCl (Sigma), 100% NP40 (Sigma), 100 mM EDTA (Sigma), 50% glycerol (Sigma), and protease inhibitor (Sigma) and then centrifuged at 10,000 x *g* for 15 min. Cell extracts were quantified using the BCA protein assay kit and stored at -80°C. Protein A/G PLUS-Agarose (Santa Cruz) was incubated with 2 *μ*g *β*-arrestin2 and CXCR6 antibodies at 4°C for 6 h, and then, 800 *μ*g of cell extracts was additionally incubated overnight at 4°C, respectively. Cell extracts were centrifuged at 12,000 x *g* for 5 min and washed three times with lysis buffer. Immunoprecipitated samples were separated by 10% SDS-PAGE and detected by western blot analysis using anti-ubiquitin (Santa Cruz), anti-*β*-arrestin2, and anti-CXCR6 antibodies. The host of anti-ubiquitin antibody is different from that of the anti-*β*-arrestin2 and anti-CXCR6 antibodies used for immunoprecipitation.

### 2.11. ELISA

Soluble CXCL16 concentrations in supernatants of hADMSCs or differentiated cells (Ad, Os) were determined by using a human CXCL16 ELISA kit (RayBiotech, USA) according to the manufacturer's instructions.

### 2.12. Inhibition of De Novo Synthesis of Protein and ADAM Activity

De novo synthesis of receptors was identified by treatment with cycloheximide (CHX; Sigma). Cells were cultured in adipogenic or osteogenic induction media with 1 *μ*g/ml CHX at the early stage of differentiation for 4 days and without CHX for 14 days, and then, CXCR6 expression was evaluated by ICC analysis. The expression of the transmembrane-bound form of CXCL16 via inhibition of ADAM activity was also measured by ICC analysis when hADMSCs were induced to adipogenic or osteogenic induction media with ADAM10 inhibitor (GI254053x; 20 *μ*M, Sigma) for 18 days.

### 2.13. Statistical Analysis

GraphPad Prism 5.0 (GraphPad Software Inc., USA) was used for all statistical analyses. Results were expressed as mean ± standard error of at least three independent experiments, and *N* numbers for experiments are described in each figure. Multiple comparisons were identified by one-way ANOVA and Bonferroni test. Comparisons of the two samples were assessed by Student's *t*-test.

## 3. Results

### 3.1. Differentiation of Adipocytes and Osteoblasts from hADMSCs

To acquire the phenotypical and molecular characteristics of differentiated cells from hADMSCs as the stage of differentiation, hADMSCs were induced to adipocytes and osteoblasts from hADMSCs using the previously reported differentiation protocol [27, 28]. First, the phenotypical changes during adipogenesis were examined by ORO staining, which stains the cellular lipid droplets. The level of ORO staining was significantly increased by ~2- and ~5-fold during adipogenic induction at day 12 and 18, respectively (Supplementary Fig. 1A). Next, extracellular calcium depositions during osteogenesis were investigated by ARS staining as well. The extracellular calcium deposition was significantly increased by ~25- and ~40-fold after 12 and 18 days from osteogenic induction, respectively (Supplementary Fig. 1B). In addition, qRT-PCR results demonstrated that the gene expression level of specific markers for adipocytes (*PPARγ*, *FABP4*, and *adiponectin*) and osteoblasts (*RUNX2*, *ALP*, and *OCN*) was significantly upregulated after 12 and 18 days of induction (Supplementary Fig. 1C-D).

### 3.2. CXCR6 Expression on the Cell Surface of hADMSCs and Differentiated Adipocytes or Osteoblasts

**Figure 1:**
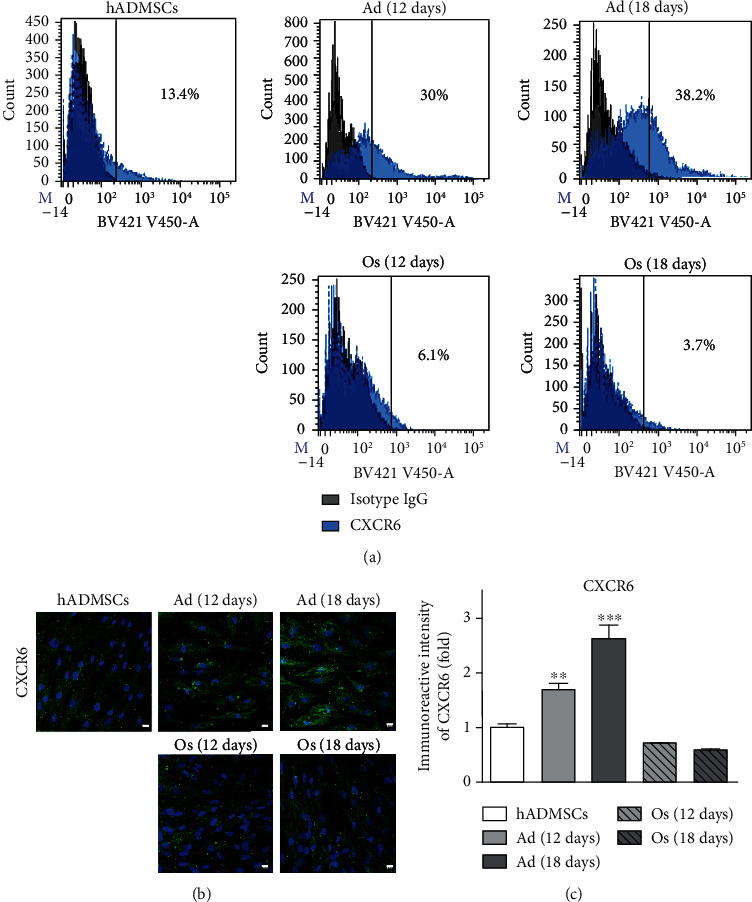
CXCR6 expression on the cell surface of hADMSCs and differentiated adipocytes and osteoblasts. (a) Flow cytometry analysis of CXCR6 (blue line) on hADMSCs, Ad (12 days), Ad (18 days), Os (12 days), and Os (18 days). The negative control of each sample is isotype IgG (black line). (b) CXCR6 expression on the cell surface was evaluated by ICC. Cells were stained with CXCR6 (green), and nuclei were stained with DAPI (blue). Scale bar = 10 *μ*m. (c) Quantification of fluorescent intensities of CXCR6 was analyzed by the ImageJ program. *N* = 5 trial per samples and control. ^∗∗^*P* < 0.01 and ^∗∗∗^*P* < 0.001 indicate statistically significant compared with the hADMSC group.

It is well known that hADMSCs express CXCR6 on the cell surface to regulate cell homing to injured sites on interaction with a specific ligand [6, 9]. As shown in Figure 1, we also confirmed that CXCR6 was expressed on hADMSCs as similarly with previous studies [6]. Interestingly, we identified a difference in the CXCR6 expression between hADMSCs and differentiated cells (Ad, Os). The flow cytometry results showed that the percentage of CXCR6-positive cells in Ad (12 days) and Ad (18 days) was increased by ~2.2- and ~2.9-fold, respectively, whereas that in Os (12 days) and Os (18 days) showed lower (×2.2 and ×3.6, respectively) than hADMSCs (Figure 1(a)). The level of CXCR6 expression was predominantly higher (×10) in Ad than Os. In addition, immunostaining results with CXCR6 antibody demonstrated that the expression level of CXCR6 was gradually increased in Ad but decreased in Os (Figures 1(b) and 1(c)). These results suggest that the CXCR6 expression might be important in regulating cell differentiation due to the reciprocal expression of CXCR6 in adipocytes and osteoblasts differentiated from hADMSCs.

### 3.3. The Effect of *CXCR6* Knockdown on the Capacity of hADMSCs to Differentiate into Adipogenic and Osteogenic Lineages

**Figure 2:**
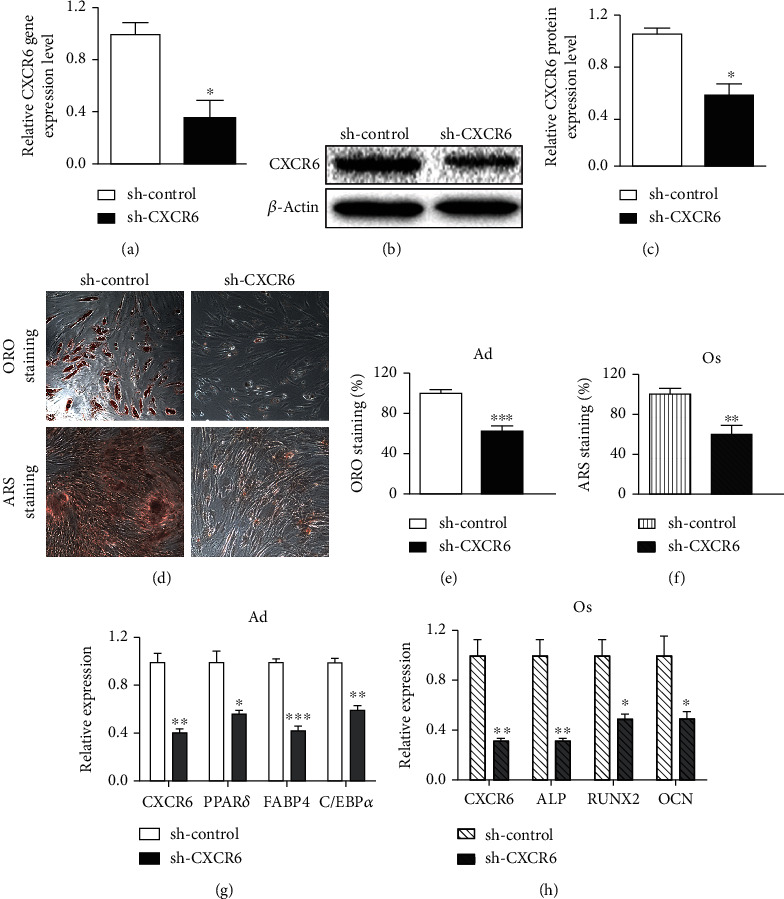
*CXCR6* knockdown by small hairpin RNA technique. (a) Quantitative *CXCR6* gene expression and (b, c) protein intensity of CXCR6. *N* = 3 trial per samples and control. The effect of CXCR6 knockdown on the capacity of hADMSC differentiation into adipogenic and osteogenic lineages by (d) ORO and ARS staining after 18 days of adipogenic and osteogenic induction. Magnification (×200). (e, f) Stained lipids and osteoblasts were dissolved and quantified. *N* = 6 trial per samples and control. (g, h) Gene expression levels of adipogenic markers (*PPARγ*, *FABP4*, and *C/EBPα*) and osteogenic markers (*ALP*, *RUNX2*, and *OCN*) on Ad and Os transfected with sh-control and sh-CXCR6. *N* = 3 trial per samples and control. ^∗^*P* < 0.05, ^∗∗^*P* < 0.01, and ^∗∗∗^*P* < 0.001 indicate statistically significant compared with the sh-control group.

To examine whether CXCR6 can affect the differentiation potential of hADMSCs, we used lentiviral particles containing human CXCR6 shRNA (sh-CXCR6) and transduced to hADMSCs to knock down CXCR6. Control hADMSCs were transduced with control shRNA lentiviral particles (sh-control). As a result of CXCR6 shRNA transduction, the expression level of *CXCR6* on hADMSCs was substantially reduced by ~2-fold compared with sh-control (Figure 2(a)). The reduced level of CXCR6 protein was also confirmed by western blot analysis (Figures 2(b) and 2(c)). Recent studies using lentivirus technology reported a similar knockdown efficiency, showing hMSCs having about 40-50% reduction of the target gene ([29, 30]. It indicates that the efficiency with lentivirus is typically lower in MSCs than that of other cells [31]. Subsequently, hADMSCs transduced with sh-CXCR6 were differentiated into Ad and Os to examine their differentiation potential. Interestingly, the differentiation capability of hADMSCs transduced with sh-CXCR6 was significantly impaired (×1.5) in comparison with hADMSCs transduced with sh-control as indicated by ORO and ARS staining (Figures 2(d)–2(f)). The gene expression level of specific markers for Ad and Os was also significantly decreased by a factor of ~2.5 (Figures 2(g) and 2(f)). These results indicate that CXCR6 is required for differentiating hADMSCs into Ad and Os.

### 3.4. The Effect of *CXCR6* Overexpression on the Capacity of hADMSCs to Differentiate into Adipogenic and Osteogenic Lineages

**Figure 3:**
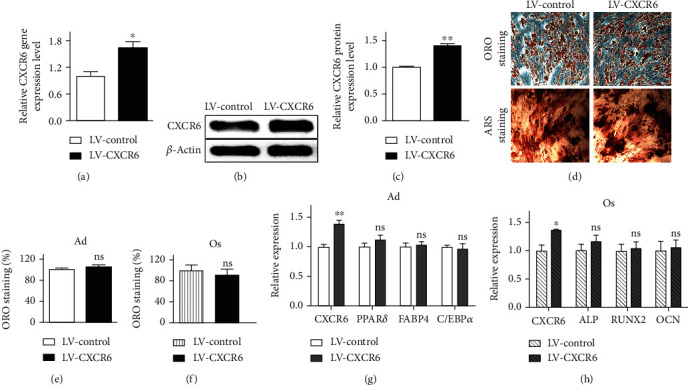
*CXCR6* overexpression by lentivirus transfection technique. (a) Quantitative *CXCR6* gene expression and (b, c) protein intensity of CXCR6. *N* = 3 trial per samples and control. The effect of LV-CXCR6 on the capacity of hADMSC differentiation into adipogenic and osteogenic lineages by (d) ORO and ARS staining after 18 days of adipogenic and osteogenic induction. Magnification (×200). (e, f) Stained lipids and osteoblasts were dissolved and quantified. *N* = 7 trial per samples and control. (g, h) Gene expression levels of adipogenic markers (*PPARγ*, *FABP4*, and *C/EBPα*) and osteogenic markers (*ALP*, *RUNX2*, and *OCN*) on Ad and Os transfected with LV-control and LV-CXCR6. *N* = 3 trial per samples and control. ^∗^*P* < 0.05 and ^∗∗^*P* < 0.01 indicate statistically significant compared with the LV-control group. ns: not significant.

In order to study the effect of CXCR6 overexpression on hADMSCs in regard to their differentiation capability, we produced lentiviral particles containing either control GFP (LV-control) or CXCR6 expression vector (LV-CXCR6) and transduced them to hADMSCs. As shown in Figures 3(a)–3(c), *CXCR6* expression in hADMSCs transduced with LV-CXCR6 was increased by ~1.6-fold compared with LV-control in both gene and protein levels. Next, we investigated the effects of CXCR6 overexpression on hADMSC differentiation. LV-CXCR6-transduced hADMSCs showed no significant difference in differentiation potential into Ad and Os compared with LV-control-transduced cells (Figures 3(d)–3(f)). Gene expression levels of adipogenic or osteogenic differentiation markers between LV-CXCR6 and LV-control also showed similar to each other (Figures 3(g) and 3(f)). It suggests that the efficiency of hADMSC differentiation is not promoted by the increase of CXCR6 expression in hADMSCs, although CXCR6 expression in hADMSCs is an important factor for the differentiation into Ad and Os.

### 3.5. Regulation of Differentiation into Adipocytes and Osteoblasts with Additional CXCL16 Treatment

**Figure 4:**
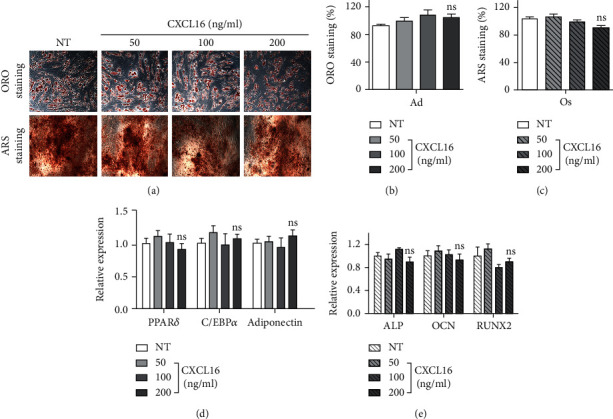
Regulation of adipogenic or osteogenic differentiation with additional CXCL16 treatment. (a) ORO and ARS staining of adipogenic-differentiated cells (Ad) and osteogenic-differentiated cells (Os) by additional CXCL16 treatment for 18 days. Percentage of (b) ORO staining on Ad and (c) ARS staining on Os. *N* = 8 trial per samples and control. Gene expression levels of (d) adipogenic markers (*PPARγ*, *C/EBPα*, and *adiponectin*) on Ad and (e) osteogenic markers (*ALP*, *OCN*, and *RUNX2*) on Os by additional CXCL16 treatment for 18 days. *N* = 3 trial per samples and control. ns: not significant.

Since overexpression of CXCR6 did not show any effects on enhancing hADMSC differentiation, we reasoned whether CXCR6 activation by CXCL16 stimulation improves the hADMSC differentiation. Thus, we additionally treated CXCL16 (200 ng/ml; PeproTech, Korea) into hADMSCs and evaluated their differentiation efficiencies by ORO and ARS staining (Figures 4(a)–4(c)). These results showed that the additional CXCL16 treatment had no significant effect on their differentiation into Ad and Os and there were no significant differences in the gene expression level of specific markers between CXCL16-treated hADMSCs and untreated control (Figures 4(d) and 4(e)). It suggests that the CXCR6 reciprocally expressed on differentiated adipocytes or osteoblasts is not for the role in regulating cell differentiation.

### 3.6. Regulation of CXCR6 Expression by Receptor Degradation or Recycling through Ubiquitination of CXCR6 or *β*-Arrestin2

**Figure 5:**
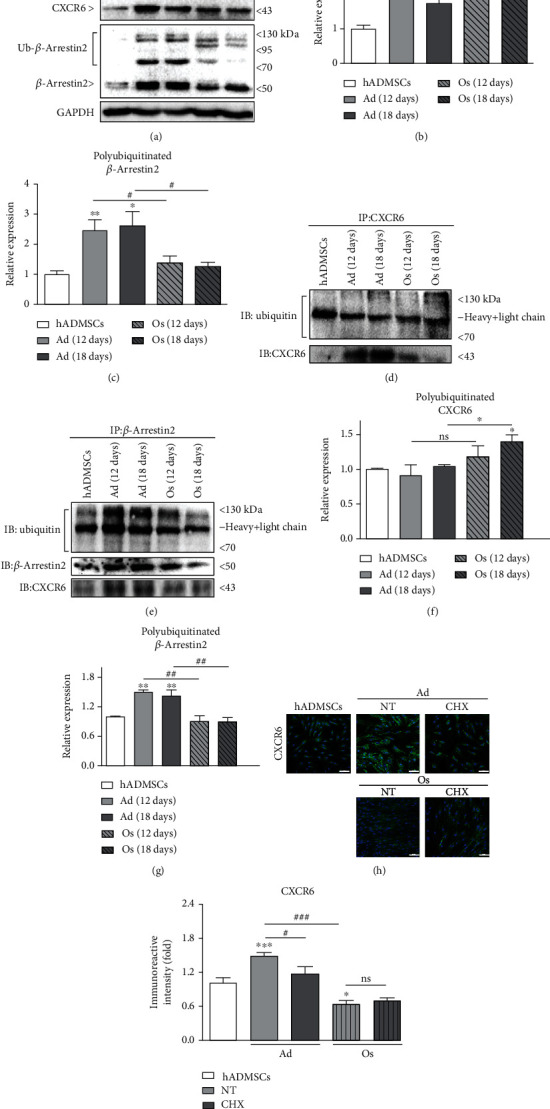
Regulation of CXCR6 degradation or recycling new synthesis in hADMSCs and differentiated adipocytes or osteoblasts. (a) Polyubiquitinated CXCR6 and *β*-arrestin2 have a greater molecular weight than that of the native proteins (44 and 50 kDa), respectively, as detected by western blot analysis. Quantitative intensities of polyubiquitination of (b) CXCR6 and (c) *β*-arrestin2. *N* = 6 trial per samples and control. (d, e) Whole cell lysate from hADMSCs, Ad (12 days), Ad (18 days), Os (12 days), and Os (18 days) was immunoprecipitated using anti-CXCR6 and anti-*β*-arrestin2 antibody and immunoblotted against ubiquitin, CXCR6, and *β*-arrestin2, respectively. Normalized intensities of (f) polyubiquitinated CXCR6 and (g) *β*-arrestin2. *N* = 3 trial per samples and control. (h) CXCR6 expression on cell surface treated with CHX (1 *μ*g/ml) for 4 days at the early stages of differentiation was evaluated by ICC. Cells were stained with CXCR6 (green), and nuclei were stained with DAPI (blue). Scale bar = 100 *μ*m. (i) Quantitative fluorescence of CXCR6 on hADMSCs, Ad, and Os treated with CHX. *N* = 5 trial per samples and control. ^∗^*P* < 0.05, ^∗∗^*P* < 0.01, and ^∗∗∗^*P* < 0.001 indicate statistically significant compared with the hADMSC group. ^#^*P* < 0.05, ^##^*P* < 0.01, and ^###^*P* < 0.001 indicate statistically significant. ns: not significant.

Chemokine receptors, which are activated by specific chemokine, are internalized and degraded by polyubiquitination of the receptor or recycled back to the plasma membrane by polyubiquitination of *β*-arrestin2 [21, 32, 33]. In this study, the ubiquitination level of CXCR6 and *β*-arrestin2 was examined by western blot and immunoprecipitation analysis to evaluate which mechanisms regulate the level of CXCR6 expression on the cell surface. First, the higher molecular weight of the target protein was detected to determine a modified protein by ubiquitination [24]. As shown in Figure 5(a), the polyubiquitinated CXCR6 had a greater molecular weight than that of the native protein (44 kDa), in differentiated cells. The normalized intensity of the higher molecular mass bands relative to native CXCR6 showed no significant difference between Ad and Os, but it was slightly increased in Os (Figure 5(b)). Conversely, polyubiquitinated *β*-arrestin2 was significantly detected at a higher molecular mass than native *β*-arrestin2 (50 kDa) in Ad, as compared with Os (Figures 5(a) and 5(c)). It means that the internalized CXCR6 was degraded in Ad, particularly in Os, but the CXCR6 recycling through *β*-arrestin2 polyubiquitination was higher (×2) in Ad than Os. Next, we performed immunoprecipitation of each protein to investigate the ubiquitination of CXCR6 and *β*-arrestin2. There was a significant increase of CXCR6 polyubiquitination in Os compared with Ad (Figures 5(d) and 5(f)). Polyubiquitinated *β*-arrestin2 was significantly increased by ~1.7-fold in Ad compared with Os, in accordance with the data in Figures 5(a) and 5(c). *β*-Arrestin2 and CXCR6 expression were also upregulated in Ad immunoprecipitated with *β*-arrestin2, indicating that CXCR6 formed a complex with polyubiquitinated *β*-arrestin2 and was destined for recycling. These results proved that higher CXCR6 expression in Ad is because it is highly recycled back to the cell membrane rather than degraded by CXCR6 polyubiquitination.

### 3.7. De Novo Synthesis of CXCR6 on the Cell Surface

The level of newly synthesized CXCR6 was investigated by using CHX, which is an inhibitor of receptor transport to the cell surface [34]. CHX (1 *μ*g/ml) treatment was applied at the early differentiation stages into adipocytes and osteoblasts for 4 days. There was no significant difference in CXCR6 expression between Os and Os treated with CHX (Figures 5(h) and 5(i)), and so it seemed unlikely that protein synthesis was involved in a newly synthesized receptor of Os. However, CXCR6 expression was significantly decreased in Ad with CHX treatment, indicating that the reason that CXCR6 was highly expressed in Ad was because of de novo synthesis as well as recycling back to the cell surface. These three mechanisms regulating CXCR6 expression are induced by CXCR6 internalization in response to its interaction with CXCL16 [15]. Therefore, we further evaluated the CXCL16 expression or secretion level in hADMSCs and differentiated cells to examine the autocrine effects of CXCL16.

### 3.8. Regulation of a Transmembrane-Bound or Soluble Form of CXCL16 in hADMSCs and Differentiated Adipocytes or Osteoblasts by ADAM Activity

**Figure 6:**
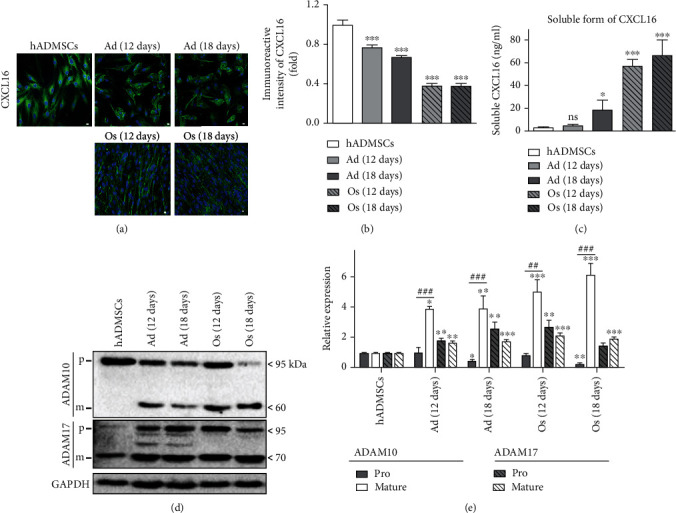
CXCL16 cleavage by ADAM10 activation in hADMSCs and differentiated adipocytes or osteoblasts. (a) Expression of transmembrane-bound CXCL16 on the cell surface was evaluated by ICC. Cells were stained with CXCL16 (green), and nuclei were stained with DAPI (blue). Scale bar = 10 *μ*m. (b) Normalized data obtained by using the ImageJ program. *N* = 6 trial per samples and control. (c) Level of soluble CXCL16 in the cell supernatant of hADMSCs, Ad (12 days), Ad (18 days), Os (12 days), and Os (18 days) was examined by ELISA. *N* = 6 trial per samples and control. (d) Protein expression of pro- and mature form of ADAM10 and ADAM17. p: proform; m: mature form. Quantification of pro- or mature form of (e) ADAM10 and ADAM17. *N* = 4 trial per samples and control. ^∗^*P* < 0.05, ^∗∗^*P* < 0.01, and ^∗∗∗^*P* < 0.001 indicate statistically significant compared with the hADMSC group. ns: not significant.

Transmembrane-bound chemokine CXCL16 undergoes metalloproteinase-dependent cleavage and is secreted in a soluble form [18]. To test the level of transmembrane-bound and soluble CXCL16, we aimed to evaluate the expression of CXCL16 on the cell surface and CXCL16 secreted in the cell culture medium of hADMSCs, Ad, and Os. The level of transmembrane-bound CXCL16 in all differentiated cells showed a significant decrease particularly in Os, whereas the level of soluble CXCL16 was inversely increased by ~2.5- and 12-fold in Ad and Os as compared with hADMSCs, respectively (Figures 6(a)–6(c)). These results indicate that transmembrane-bound CXCL16 in hADMSCs was significantly cleaved and secreted in the soluble form during the differentiation of cells into Ad and Os. ADAM proteins, which cleave transmembrane-bound CXCL16, exist as proform zymogens and only exhibit sheddase activities once proteolytically cleaved and activated [15, 35]. To confirm the effect of cleavage on CXCL16 by ADAM, we assessed the expression of the form (pro and mature) of two members of the ADAM family (ADAM10 and ADAM17) on hADMSCs, Ad, and Os by western blot analysis. Pro-ADAM10 or pro-ADAM17 (100 kDa) was cleaved to the active mature-protease form (60 or 70 kDa, respectively). Particularly, ADAM10 was significantly activated by ~3.9-, 8.7-, 6.3-, and 24.5-fold compared with pro-ADAM10 in Ad (12 days), Ad (18 days), Os (12 days), and Os (18 days), respectively (Figures 6(d) and 6(e)). The activation level of ADAM17 was also activated, but the pro- and mature form in the cells did not significantly differ from each other (Figure 6(e)). It indicates that CXCL16 that exists in transmembrane bound in hADMSCs was cleaved and secreted as the soluble form by ADAM10 activation in both differentiated cells, notably in Os. Moreover, we treated ADAM10 inhibitor (GI24023x; 20 *μ*M), which inhibits the shedding of CXCL16, to clarify the effects of ADAM10 activity on the cleavage of CXC16. The level of transmembrane-bound CXCL16 in Os treated with GI24023x was significantly increased (×1.8) by a similar level as that of hADMSCs. Ad treated with GI24023 showed a slight increase (×1.3) in transmembrane-bound CXCL16 (Figures 6(a) and 6(b)). These results correlate with the data in Figures 6(d) and 6(e), suggesting that CXCL16 is cleaved by ADAM10 and secreted as a soluble form in Ad and Os, in particular.

### 3.9. CXCR6 Expression on Differentiated Adipocytes and Osteoblasts by Inhibition of CXCL16 Secretion

**Figure 7:**
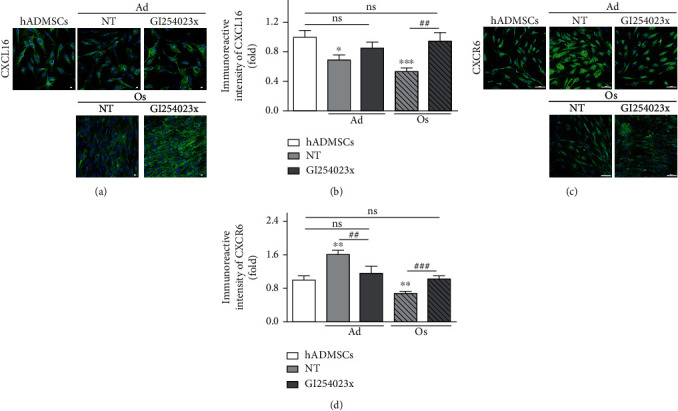
Level of CXCL16 secretion and CXCR6 expression by inhibition of ADAM10 activity in hADMSCs and differentiated adipocytes or osteoblasts. (a) Expression of transmembrane-bound CXCL16 in cells treated with ADAM10 inhibitor (GI254023x; 20 *μ*M) was evaluated by ICC. Cells were stained with CXCL16 (green), and nuclei were stained with DAPI (blue). Scale bar = 10 *μ*m. (b) Quantification of fluorescent intensities of CXCL16 was performed by the ImageJ program. *N* = 6 trial per samples and control. (c) CXCR6 expression on the surface of cells treated with GI254023x was evaluated by ICC. Cells were stained with CXCR6 (green), and nuclei were stained with DAPI (blue). Scale bar = 100 *μ*m. (d) Fluorescent intensities of CXCR6 were quantified by the ImageJ program. *N* = 6 trial per samples and control. ^∗^*P* < 0.05 and ^∗∗^*P* < 0.01 indicate statistically significant compared with the hADMSC group. ^##^*P* < 0.01 and ^###^*P* < 0.001 indicate statistically significant between the NT- and GI254023x-treated groups in Ad or Os. ns: not significant.

Additionally, the expression of CXCR6 on differentiated cells (Ad and Os) treated with GI24023x was evaluated to demonstrate that the level of soluble CXCL16 directly regulating CXCR6 expression. These results may substantially prove the molecular mechanism whereby soluble CXCL16 secreted by ADAM10 binds to CXCR6, which then modulates the intracellular response for internalization. As shown in Figures 7(c) and 7(d), CXCR6 expression on Ad and Os was significantly increased and decreased, respectively, in line with the data shown in Figure 1. However, Ad and Os treated with GI254023x showed significantly decreased and increased CXCR6 expression, respectively, showing a similar level with that of in hADMSCs. It means that CXCL16 secreted by ADAM10 binds to CXCR6, which is, in turn, internalized and regulates CXCR6 expression on the cell surface. It is known that cleaved CXCL16-mediated internalization leads to the loss of CXCR6 from the cell surface [15]. As evident from that report, it can be inferred from our results that the internalization of CXCR6 by interaction with highly secreted CXCL16 occurs to a greater extent in Os than Ad. Taken together, our data suggest that Os exhibit lower CXCR6 expression than hADMSCs as a result of the CXCR6 internalization by interaction with the relatively high secretion of CXCL16 during the differentiation into Os, and the internalized CXCR6 is slightly degraded rather than recycled. In contrast, Ad exhibit higher CXCR6 expression than hADMSCs due to the CXCR6 internalization by interaction with the relatively low secretion of CXCL16 during the differentiation into Ad, and the internalized receptor is highly recycled and newly synthesized rather than degraded. In conclusion, this study may provide the molecular mechanism underlying CXCR6 expression that is reciprocally expressed on the differentiated cells by the regulation of CXCR6 degradation or recycling in response to prolonged stimulation with CXCL16 and also by de novo synthesis of the receptor.

## 4. Discussion

Chemokine receptors play crucial roles in physiological cellular processes associated with various inflammatory or immunological diseases by interaction with specific chemokine [36–38]. Chemokine receptors are mainly expressed on leukocytes, but hADMSCs expressing chemokine receptors can be used for promising applications in the field of regenerative medicine owing to their ability to regulate cell migration or engraftment to affected tissues [2, 4, 9]. Particularly, CXCR6 expressed on the stem cell surface is important for migration and recruitment to sites of damaged tissue. However, the investigation on the expression of CXCR6 in Ad and Os from hADMSCs and the molecular mechanism remains unclear.

In this study, we focused on how the CXCR6 expression was reciprocally regulated in Ad and Os and what is the role of CXCR6 highly expressed in Ad in particular (Figure 1). As shown in Figures 2(d)–2(f), the efficiency of differentiation into Ad and Os from hADMSCs transduced with sh-CXCR6 was lower (×2) relative to control. The gene expression level of specific markers for Ad and Os was also significantly decreased in cells transduced with sh-CXCR6 (Figures 2(g) and 2(h)). It indicates that CXCR6 expression on hADMSCs is one of the important factors directing differentiation into Ad and Os. Notably, hADMSCs overexpressing CXCR6 (LV-CXCR6) showed no significant difference in the differentiation potential of hADMSCs as compared with LV-control (Figures 3(d)–3(h)). These results suggest that the increased expression of CXCR6 on cell surface does not act as an enhancer for the differentiation of hADMSCs but it is necessary for the differentiation of hADMSCs. As shown in Figure 4, the efficacy of the differentiation into Ad and Os was not affected by additional CXCL16 treatment, indicating that the CXCR6 activation also does not affect the cell differentiation irrespective of the presence or absence of CXCL16. Overall, we can conclude that the expression or activation of CXCR6 does not induce cell differentiation but is regulated during their differentiation stages.

It is known that the number of chemokine receptors on the cell surface is balanced following the fate of internalized chemokine receptor by interaction with specific chemokine [21, 22, 25]. When chemokine receptor CXCR6 is internalized by CXCL16 stimulation, recruited *β*-arrestin2 binds to the receptor and then degraded or recycled back to the plasma membrane depending on whether ubiquitin binds to which protein. Polyubiquitinated CXCR6 is dissociated from *β*-arrestin2 and degraded, whereas polyubiquitinated *β*-arrestin2 forms a complex with CXCR6, and in turn, the receptor is recycled [23]. We initially observed the polyubiquitination of CXCR6 or *β*-arrestin2 by detecting the band corresponding to the molecular weight of the modified target proteins, respectively. Our study showed there was slightly more polyubiquitinated CXCR6 in Os than Ad (Figure 5(a)), but there was no significant increase in the normalized graph (Figure 5(b)). The intensity of modified *β*-arrestin2 was significantly higher (×1.8 and ×2.1, respectively) in Ad than in Os (Figures 5(a) and 5(c)). These results indicate that CXCR6 is slightly degraded by polyubiquitination of CXCR6 in Os, but it is highly recycled back to the cell surface in Ad due to polyubiquitination of *β*-arrestin2. Cell lysates immunoprecipitated with CXCR6 showed higher CXCR6 expression in Ad, as we expected, and the receptor was significantly polyubiquitinated in Os than Ad, indicating that CXCR6 is highly degraded in Os (Figures 5(d) and 5(f)). In contrast, the expression level of polyubiquitinated *β*-arrestin2 was significantly high in Ad as compared with Os. *β*-arrestin2 or CXCR6 was also highly expressed in Ad immunoprecipitated with *β*-arrestin2 (Figures 5(e) and 5(g)). It suggests that CXCR6 is highly recycled in Ad, forming a complex with polyubiquitinated *β*-arrestin2 [23].

Besides the ubiquitination of CXCR6 or *β*-arrestin2, de novo synthesis of receptors is also one of the important factors regulating the number of receptors on the cell surface. It was proven by treatment with CHX, which is the most common method used to inhibit the protein transport of newly synthesized receptors to the plasma membrane [25, 34, 39, 40]. CXCR6 expression was significantly decreased in Ad treated with CHX as compared with Ad, while there was no significant difference in the case of Os (Figures 5(h) and 5(i)). It indicates that another mechanism for the increased level of CXCR6 expression on Ad is due to the de novo synthesis of receptors and subsequent movement to the cell surface via protein transport. These results suggest that the increased level of CXCR6 expression on Ad is due to the highly recycled CXCR6 to the cell surface by polyubiquitination of *β*-arrestin2 and de novo synthesis, rather than the degradation of CXCR6. As evident in Figure 5, we inferred that the level of CXCR6 expression was regulated by three mechanisms and it can be affected by interaction with its specific ligand, CXCL16.

Consequently, we observed the CXCL16 expression or secretion level on hADMSCs and differentiated cells to investigate the autocrine effects of CXCL16. CXCL16 is bound to the membrane, and it is cleaved to a soluble form by activation of ADAM10, which is the most prevalent protease for the constitutive CXCL16 shedding [15, 18, 19]. As shown in Figures 6(a) and 6(b), the expression of transmembrane-bound CXCL16 in hADMSCs was significantly decreased in both of the differentiated cells, which inversely correlated with the level of soluble CXCL16 that was increased by ~10-fold in Os as compared with the amount in the hADMSCs (Figure 6(c)). ADAM10 was significantly cleaved to the mature form (60 kDa) in Os than Ad, correlated to the results of Figures 6(a)–6(c) (Figures 6(d) and 6(e)). It indicates that CXCL16 is highly cleaved to its soluble form by ADAM10 activation in Ad, and more particularly in Os. Furthermore, the effect of ADAM10 was clarified by treatment with the selective ADAM10 inhibitor, GI254023x [15]. These results confirmed that the transmembrane-bound CXCL16 in hADMSCs was secreted as a soluble form by ADAM10 activation during the differentiation into Ad, and more particularly in Os.

Interestingly, there was no significant difference in CXCR6 expression between hADMSCs and differentiated cells (Ad and Os) upon treatment with GI254023x, inhibiting the cleavage of ADAM10 (Figures 7(c) and 7(d)). It indicates that CXCR6 expression in Ad and Os is obviously regulated by the level of soluble CXCL16 secreted in response to activated ADAM10. These results indicate that the secretion of soluble CXCL16, following activation of ADAM10, induces the CXCL16-CXCR6 interaction, which is, in turn, internalized and regulates the number of CXCR6 on the cell surface by its degradation or recycling.

CXCR6 expressed on stem cells has a crucial role in inducing cell migration to damaged cells by its interaction with CXCL16 [6]. Consequently, the relative ratio of migrated cells by CXCL16 treatment was evaluated to examine that the migratory function of CXCR6 highly expressed in Ad. As the result, hADMSCs showed migration capacity by CXCL16 treatment as recently reported [6, 9], whereas there showed no effect on cell migration capacity in Ad and Os (Supplementary Fig. 2). It is natural that differentiated cells show no change in cell migration capacity, because cells promote the property of cell adhesion when differentiating into specific lineages and, conversely, cell movement is also reduced [41]. This result suggests that the CXCR6 expression in Ad might have another cellular function except for the regulation of cell migration.

Taken together, our study investigated how the CXCR6 expression was reciprocally different between Ad and Os by elucidating the mechanism on three fates of CXCR6 and CXCL16 secretion during hADMSC differentiation. It was evident that the level of CXCR6 expression in Ad was significantly increased following the CXCR6, internalized by its interaction with soluble CXCL16 and highly recycled rather than degraded during adipogenic differentiation. In contrast, the level of CXCR6 expression in Os was decreased, following the highly internalized CXCR6 interacting with significantly secreted CXCL16 and internalized CXCR6 that was degraded, only partially recycling back to the cell surface during osteogenic differentiation. Based on this data, we may provide new information on the molecular mechanisms of the CXCR6 expression in Ad and Os.

## 5. Conclusion

In this study, we demonstrated how CXCR6 expression was regulated during hADMSC differentiation into adipocytes and osteoblasts by modulation of molecular mechanisms following the secretion level of soluble CXCL16. CXCR6 was highly expressed in adipocytes due to CXCR6 recycling when it is internalized by interaction with soluble CXCL16. In contrast, osteoblasts showed a lower CXCR6 expression due to the degradation of internalized CXCR6 as a result of a high level of soluble CXCL16. These data may provide fundamental information on the molecular mechanisms of CXCR6 reciprocally expressed on differentiated adipocytes and osteoblasts from hADMSCs. In addition, utilizing chemokine receptor and chemokine that are expressed and secreted in differentiated cells from hADMSCs may contribute to the control of the inflammatory response that can occur during the clinical implications of hADMSCs. That is, it may contribute to the enhancement of efficacy providing insights on challenges remained in stem cell therapy.

## Data Availability

The datasets used in the study are available from the corresponding author on reasonable request.

## Supplementary Materials

Supplementary Materials Supplementary Figure 1: establishment of adipogenic or osteogenic differentiation from hADMSCs. (A) ORO staining of hADMSCs and adipogenic-differentiated cells (Ad) and (B) ARS staining of hADMSCs and osteogenic-differentiated cells (Os) for 12 and 18 days. *N* = 3 trial per samples and control. The RNA expression level of (C) adipogenic markers (*PPARγ*, *FABP4*, and *adiponectin*) on hADMSCs, Ad (12 days), and Ad (18 days) and (D) osteogenic markers (*RUNX2*, *ALP*, and *OCN*) on hADMSCs, Os (12 days), and Os (18 days). *N* = 3 trial per samples and control. ^∗∗^*P* < 0.01 and ^∗∗∗^*P* < 0.001 indicate statistically significant compared with the hADMSC group. ^#^*P* < 0.05, ^##^*P* < 0.01, and ^###^*P* < 0.001 indicate statistically significant. Supplementary Figure 2: migration capacity of CXCL16 on adipogenic of osteogenic-differentiated cells from hADMSCs. (A) Migratory effects of CXCL16 on hADMSCs, adipocytes, and osteoblasts. hADMSCs and differentiated cells were scratched in the center of the dishes. Detached cells were removed by washing with PBS and placed in serum-free DMEM media (Gibco) with or without 200 ng/ml CXCL16 (PeproTech, Korea) for 48 h. Images per dish were taken under microscopy (Leica Microsystems, Germany) at 0 h and 48 h. (B) Relative ratio of migrated cells under CXCL16 treatment on differentiated cells (Ad, Os) and hADMSCs. *N* = 4 trial per samples and control. ^∗∗^*P* < 0.01 indicates statistically significant compared with the nontreated group. ns: not significant.
