# Supplementary material for: Regulation of CXCR6 Expression on Adipocytes and Osteoblasts Differentiated from Human Adipose Tissue-Derived Mesenchymal Stem Cells
Source: Stem Cells Int. 2020 Aug 19;2020:8870133. doi: 10.1155/2020/8870133 (PMC7453243; doi:10.1155/2020/8870133)
Supplement: Supplementary Materials — Supplementary Figure 1: establishment of adipogenic or osteogenic differentiation from hADMSCs. (A) ORO staining of hADMSCs and adipogenic-differentiated cells (Ad) and (B) ARS staining of hADMSCs and osteogenic-differentiated cells (Os) for 12 and 18 days. N = 3 trial per samples and control. The RNA expression level of (C) adipogenic markers (PPARγ, FABP4, and adiponectin) on hADMSCs, Ad (12 days), and Ad (18 days) and (D) osteogenic markers (RUNX2, ALP, and OCN) on hADMSCs, Os (12 days), and Os (18 days). N = 3 trial per samples and control. ∗∗P < 0.01 and ∗∗∗P < 0.001 indicate statistically significant compared with the hADMSC group. #P < 0.05, ##P < 0.01, and ###P < 0.001 indicate statistically significant. Supplementary Figure 2: migration capacity of CXCL16 on adipogenic of osteogenic-differentiated cells from hADMSCs. (A) Migratory effects of CXCL16 on hADMSCs, adipocytes, and osteoblasts. hADMSCs and differentiated cells were scratched in the center of the dishes. Detached cells were removed by washing with PBS and placed in serum-free DMEM media (Gibco) with or without 200 ng/ml CXCL16 (PeproTech, Korea) for 48 h. Images per dish were taken under microscopy (Leica Microsystems, Germany) at 0 h and 48 h. (B) Relative ratio of migrated cells under CXCL16 treatment on differentiated cells (Ad, Os) and hADMSCs. N = 4 trial per samples and control. ∗∗P < 0.01 indicates statistically significant compared with the nontreated group. ns: not significant. [file 8870133.f1.docx]

**Regulation of CXCR6 expression on adipocytes and osteoblasts differentiated from human adipose tissue-derived mesenchymal stem cells**

**Seung-Cheol Lee^a,1^, Yoo-Jung Lee^a,1^, Min Kyoung Shin, Jung-Suk Sung***

**^a^** *Department of Life Science, Dongguk University-Seoul, Goyang, Gyeonggi-do 10326, Republic of Korea*

**Supplementary figure and figure legend**

**
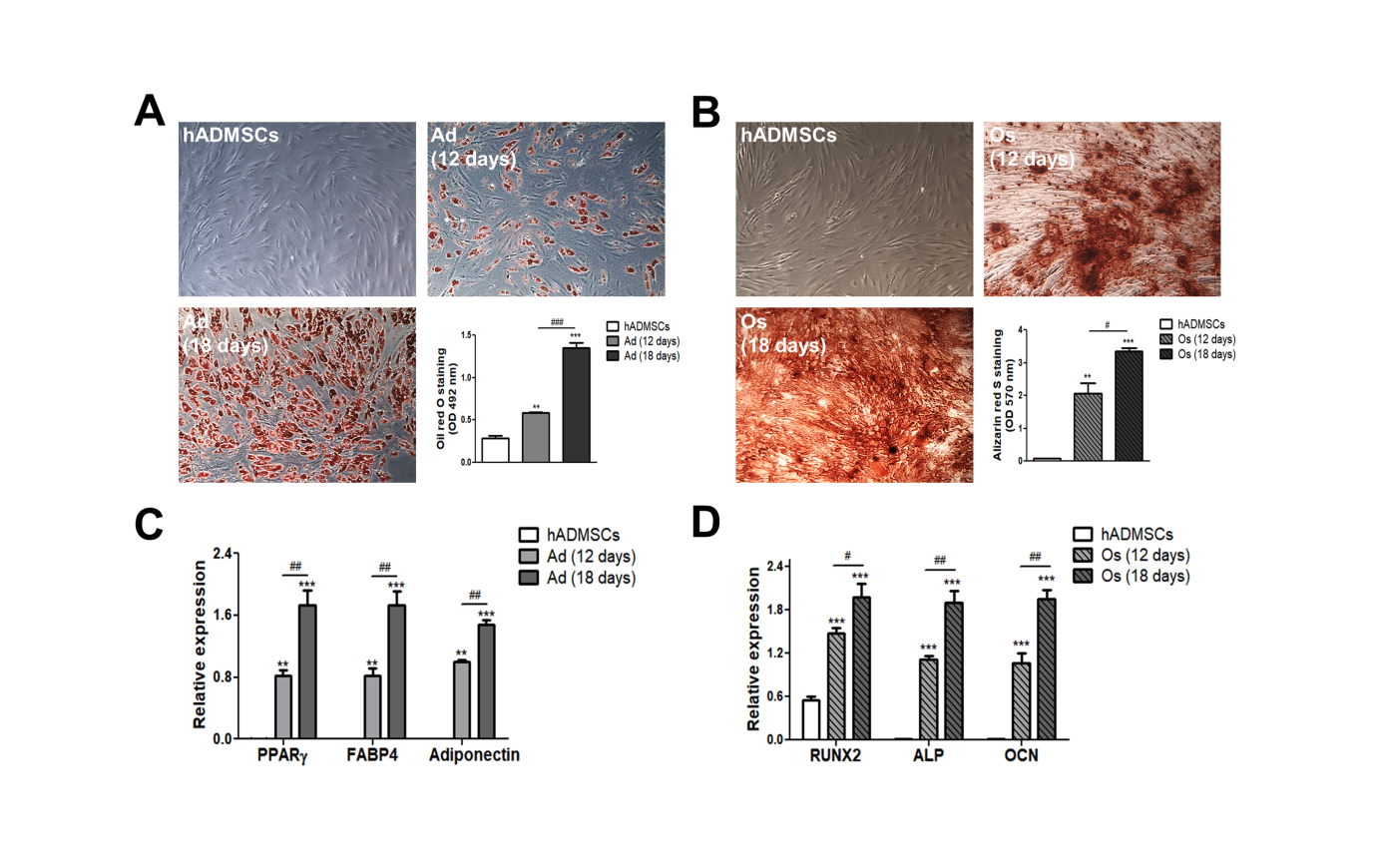
**

**Supplementary FIGURE 1: Establishment of adipogenic or osteogenic differentiation from hADMSCs** (A) ORO staining of hADMSCs and adipogenic differentiated cells (Ad) and (B) ARS staining of hADMSCs and osteogenic differentiated cells (Os) for 12 and 18 days. N=3 trial per samples and control. RNA expression level of (C) adipogenic markers (*PPARγ, FABP4,* and *adiponectin*) on hADMSCs, Ad (12 days), and Ad (18 days), and (D) osteogenic markers (*RUNX2, ALP,* and *OCN*) on hADMSCs, Os (12 days), and Os (18 days). N=3 trial per samples and control. **P<0.01 and ***P<0.001 indicate statistically significant compared with the hADMSCs group. ^#^P<0.05, ^##^P<0.01, and ^###^P<0.001 indicate statistically significant.

**
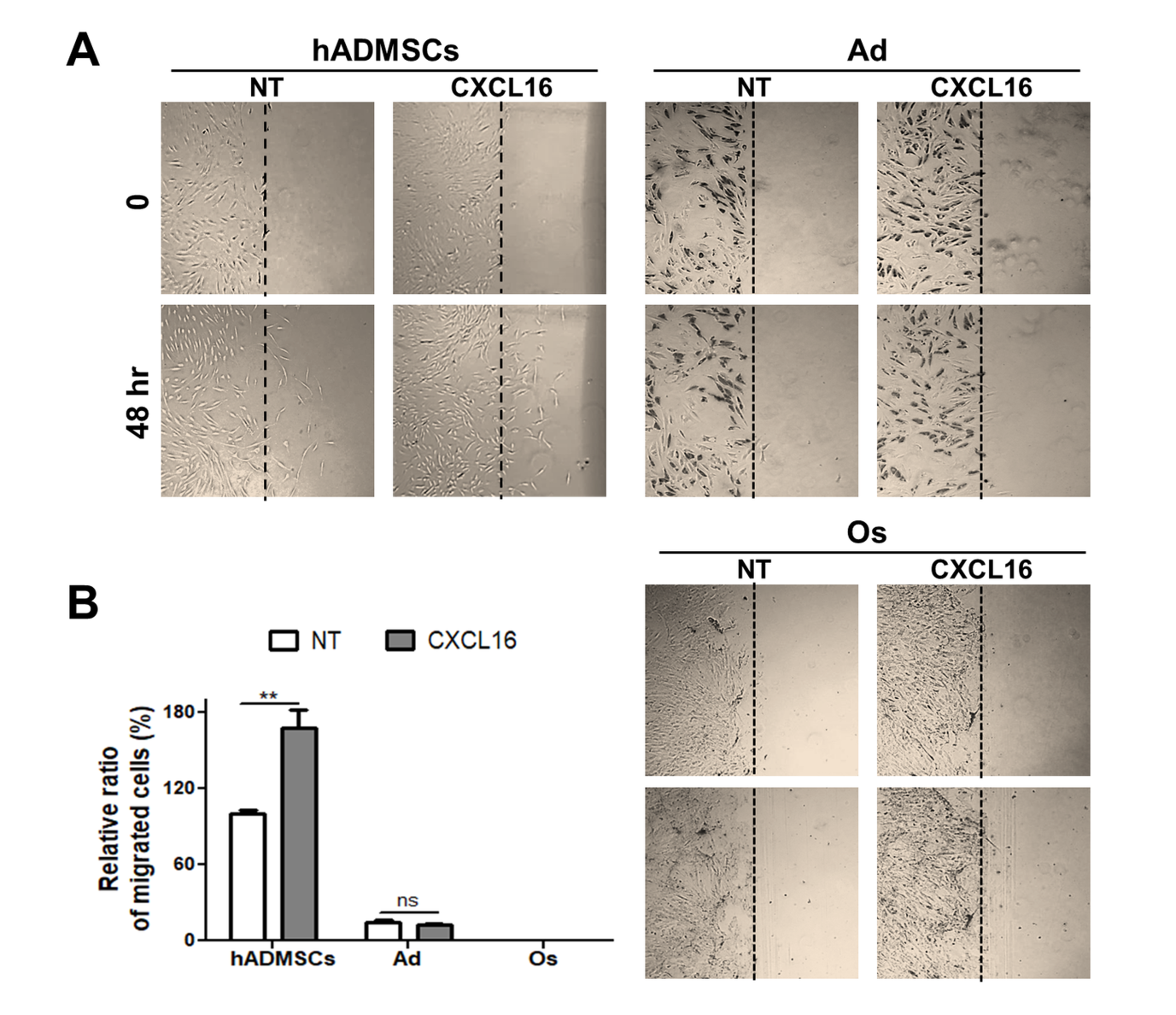
**

**Supplementary FIGURE 2: Migration capacity of CXCL16 on adipogenic of osteogenic differentiated cells from hADMSCs** (A) Migratory effects of CXCL16 on hADMSCs, adipocytes and osteoblasts. hADMSCs and differentiated cells were scratched and placed in serum-free DMEM media (Gibco) with or without 200 ng/ml CXCL16 (PeproTech, Korea) for 48 h. Images per dish were taken under microscopy (Leica Microsystems, Germany) at 0 h and 48 h. (B) Relative ratio of migrated cells under CXCL16 treatment on differentiated cells (Ad, Os) and hADMSCs. N=4 trial per samples and control. **P<0.01 indicate statistically significant compared with the Non-treated group. Not significant, ns.
